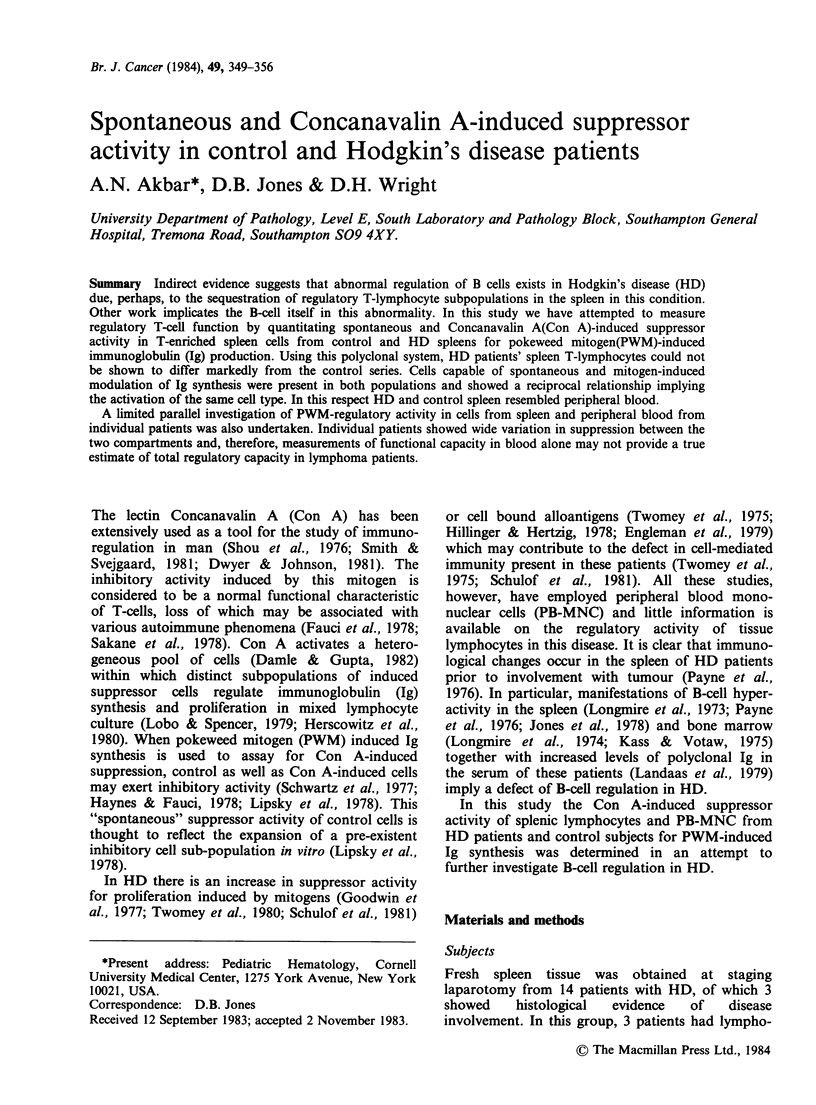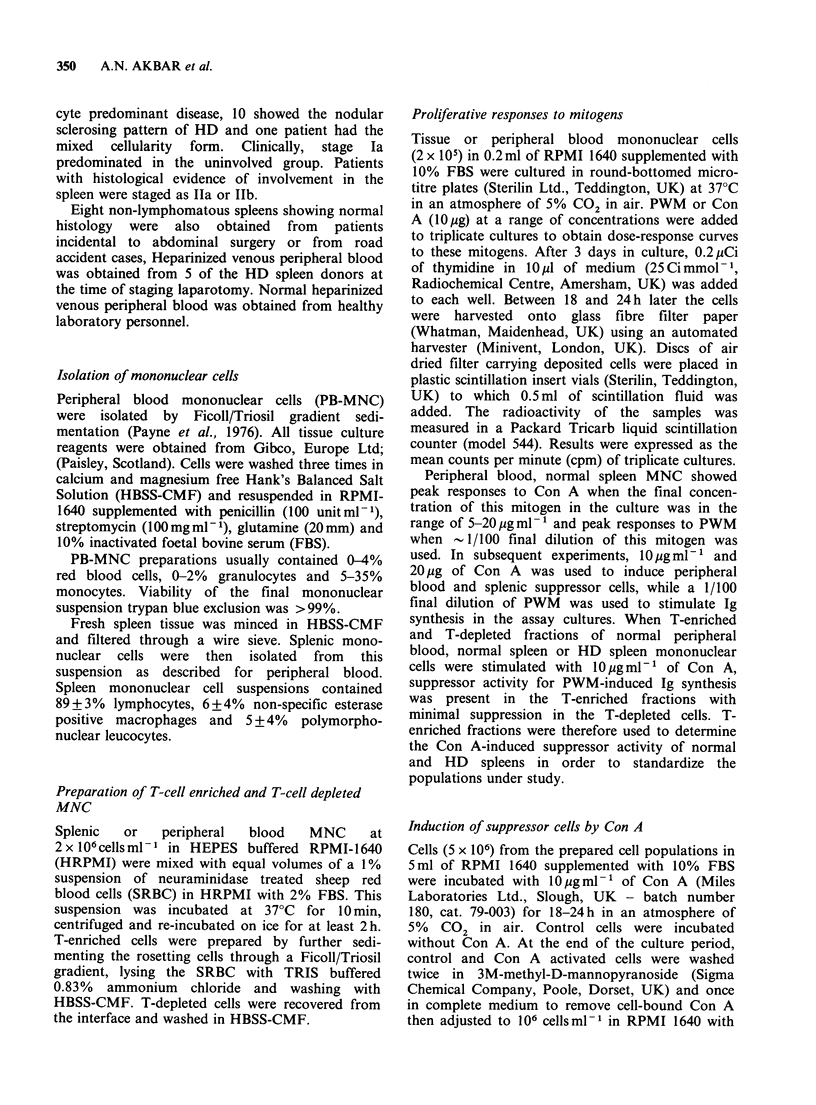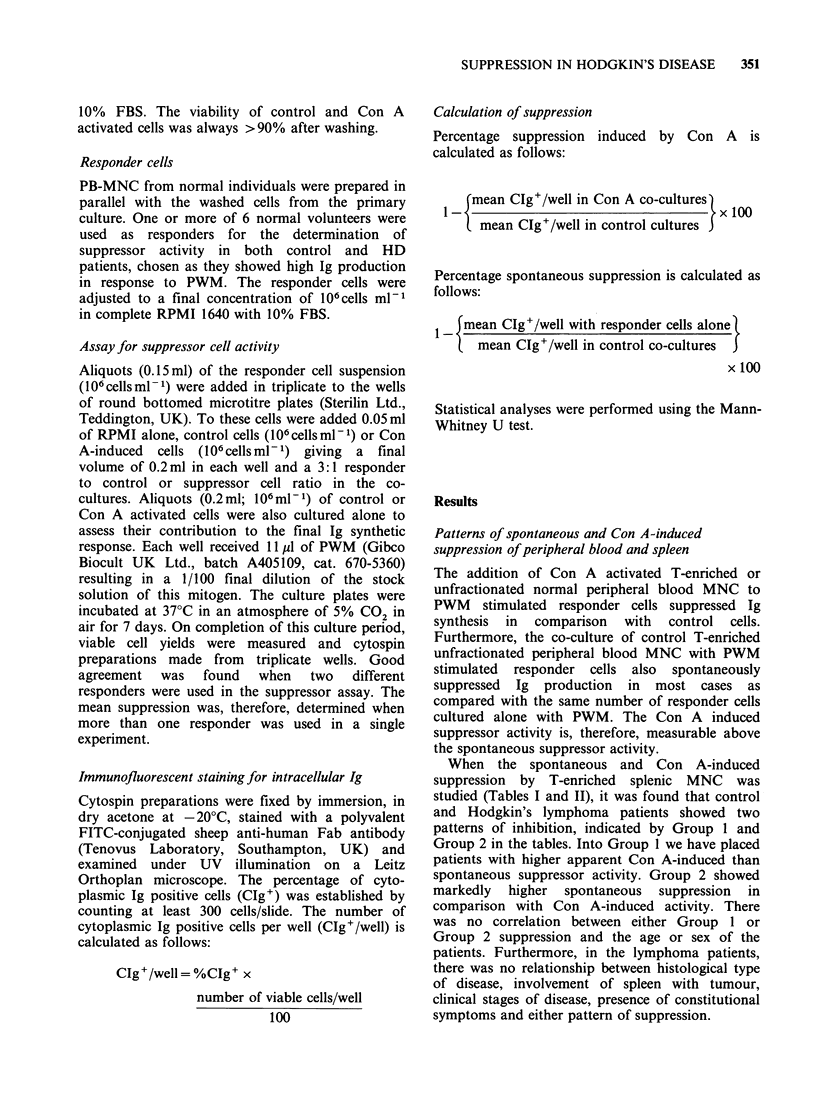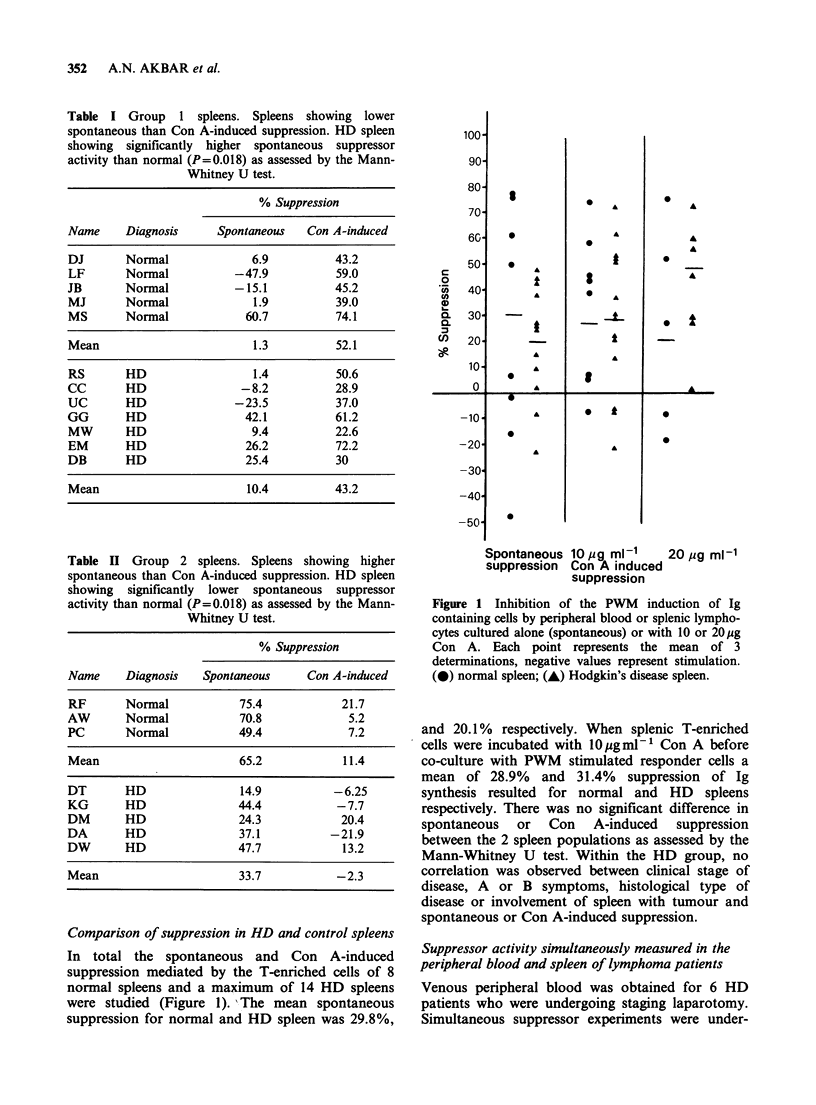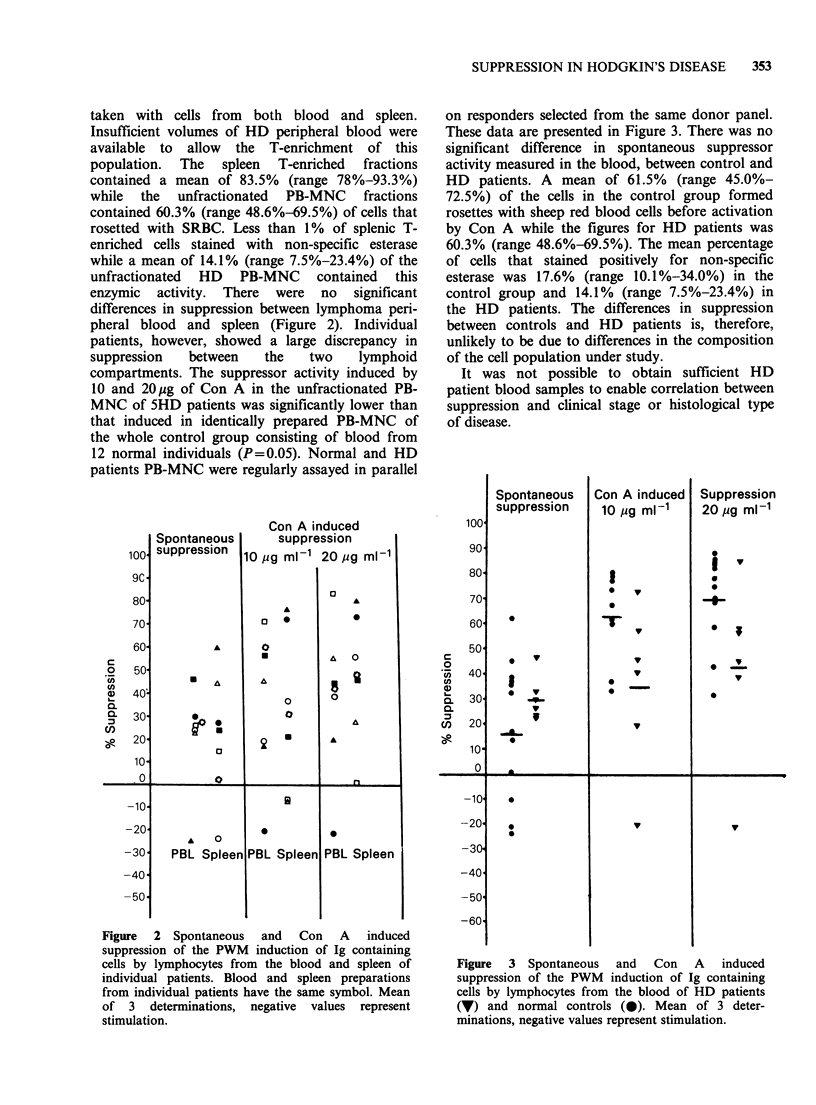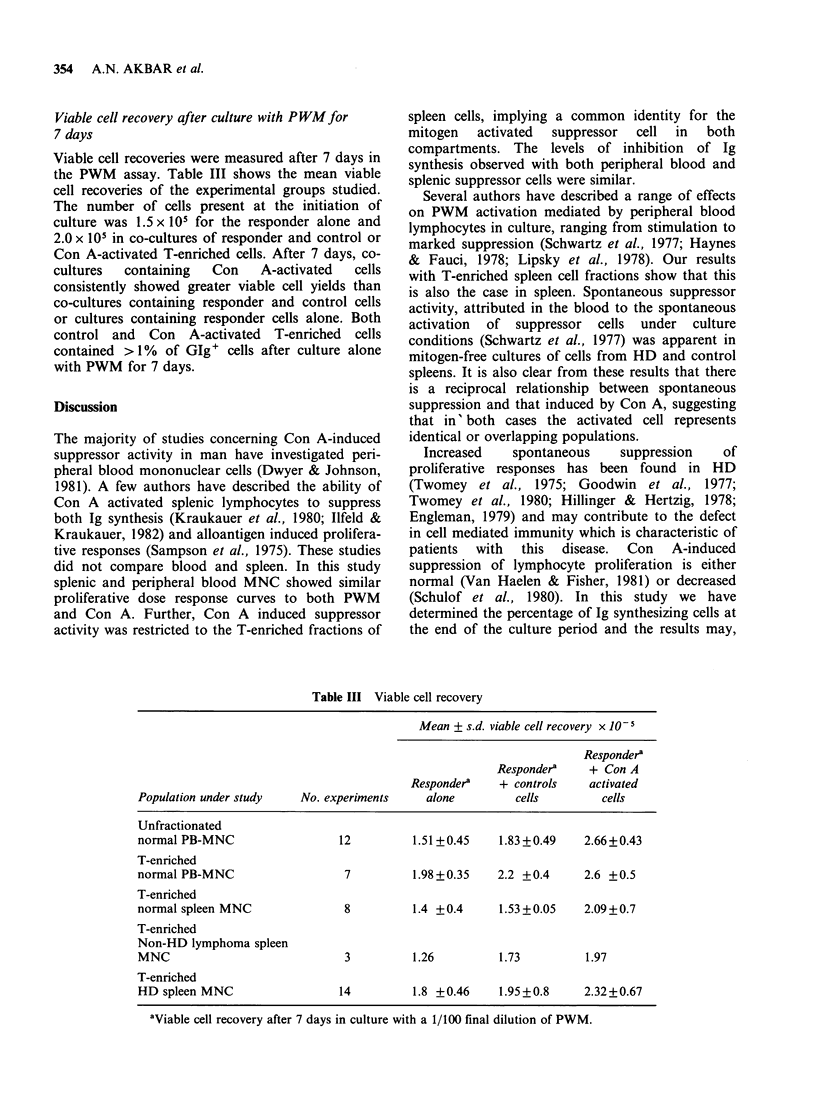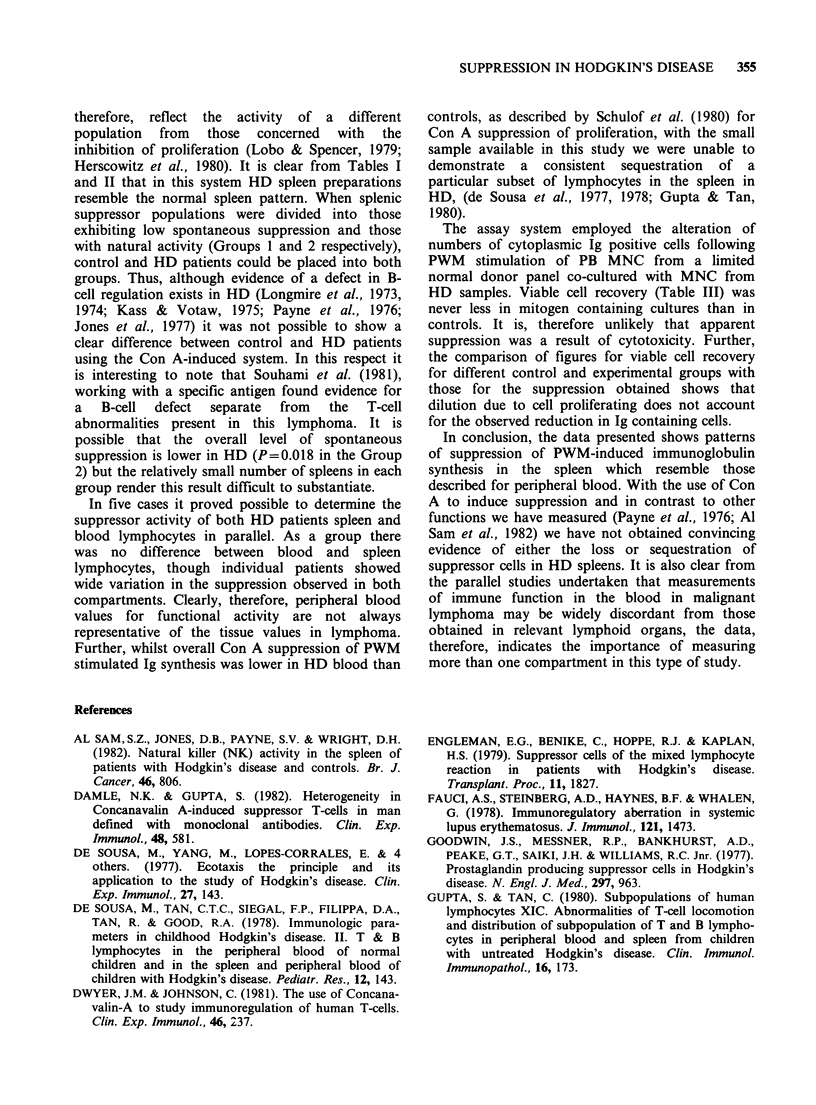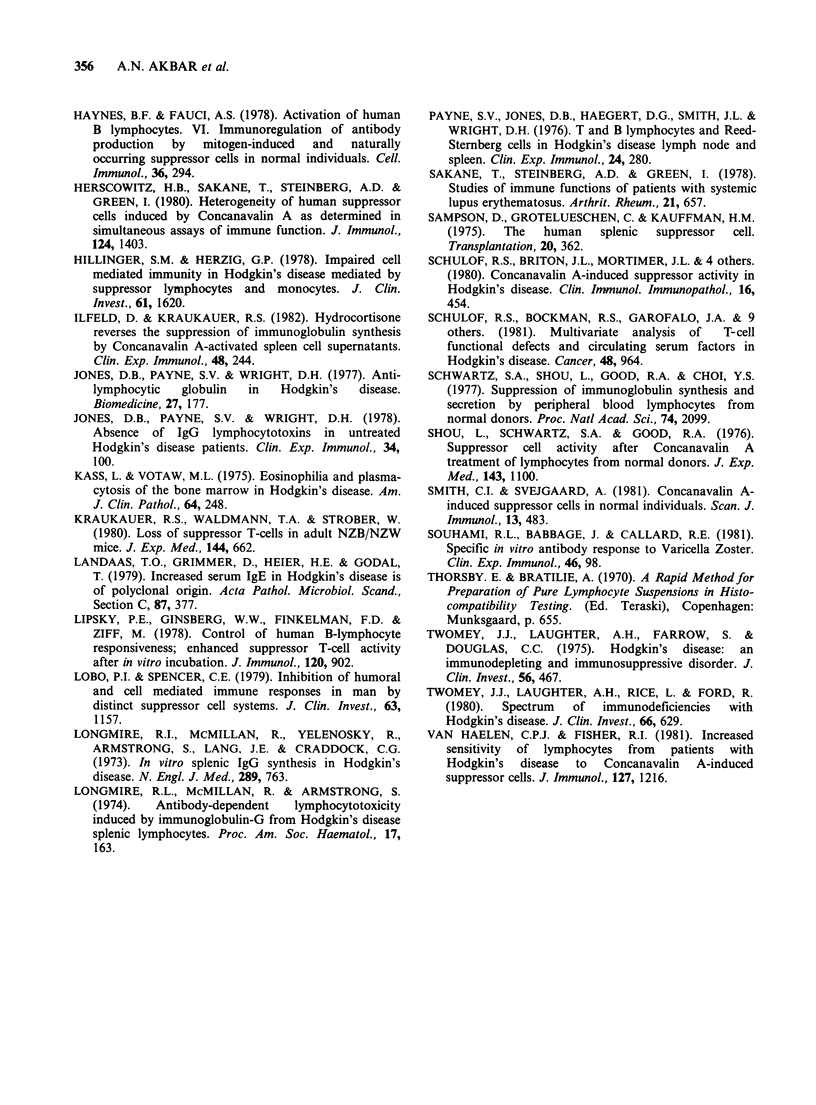# Spontaneous and Concanavalin A-induced suppressor activity in control and Hodgkin's disease patients.

**DOI:** 10.1038/bjc.1984.55

**Published:** 1984-03

**Authors:** A. N. Akbar, D. B. Jones, D. H. Wright

## Abstract

Indirect evidence suggests that abnormal regulation of B cells exists in Hodgkin's disease (HD) due, perhaps, to the sequestration of regulatory T-lymphocyte subpopulations in the spleen in this condition. Other work implicates the B-cell itself in this abnormality. In this study we have attempted to measure regulatory T-cell function by quantitating spontaneous and Concanavalin A(Con A)-induced suppressor activity in T-enriched spleen cells from control and HD spleens for pokeweed mitogen(PWM)-induced immunoglobulin (Ig) production. Using this polyclonal system, HD patients' spleen T-lymphocytes could not be shown to differ markedly from the control series. Cells capable of spontaneous and mitogen-induced modulation of Ig synthesis were present in both populations and showed a reciprocal relationship implying the activation of the same cell type. In this respect HD and control spleen resembled peripheral blood. A limited parallel investigation of PWM-regulatory activity in cells from spleen and peripheral blood from individual patients was also undertaken. Individual patients showed wide variation in suppression between the two compartments and, therefore, measurements of functional capacity in blood alone may not provide a true estimate of total regulatory capacity in lymphoma patients.


Br. J. Cancer (1984), 49, 349–356

### Spontaneous and Concanavalin A-induced suppressor activity in control and Hodgkin's disease patients

A.N. Akbar*, D.B. Jones & D.H. Wright

*University Department of Pathology, Level E, South Laboratory and Pathology Block, Southampton General Hospital, Tremona Road, Southampton SO9 4XY.*

**Summary** Indirect evidence suggests that abnormal regulation of B cells exists in Hodgkin's disease (HD) due, perhaps, to the sequestration of regulatory T-lymphocyte subpopulations in the spleen in this condition. Other work implicates the B-cell itself in this abnormality. In this study we have attempted to measure regulatory T-cell function by quantitating spontaneous and Concanavalin A(Con A)-induced suppressor activity in T-enriched spleen cells from control and HD spleens for pokeweed mitogen(PWM)-induced immunoglobulin (Ig) production. Using this polyclonal system, HD patients' spleen T-lymphocytes could not be shown to differ markedly from the control series. Cells capable of spontaneous and mitogen-induced modulation of Ig synthesis were present in both populations and showed a reciprocal relationship implying the activation of the same cell type. In this respect HD and control spleen resembled peripheral blood.

A limited parallel investigation of PWM-regulatory activity in cells from spleen and peripheral blood from individual patients was also undertaken. Individual patients showed wide variation in suppression between the two compartments and, therefore, measurements of functional capacity in blood alone may not provide a true estimate of total regulatory capacity in lymphoma patients.

The lectin Concanavalin A (Con A) has been extensively used as a tool for the study of immunoregulation in man (Shou *et al.*, 1976; Smith & Svejgaard, 1981; Dwyer & Johnson, 1981). The inhibitory activity induced by this mitogen is considered to be a normal functional characteristic of T-cells, loss of which may be associated with various autoimmune phenomena (Fauci *et al.*, 1978; Sakane *et al.*, 1978). Con A activates a heterogeneous pool of cells (Damle & Gupta, 1982) within which distinct subpopulations of induced suppressor cells regulate immunoglobulin (Ig) synthesis and proliferation in mixed lymphocyte culture (Lobo & Spencer, 1979; Herscowitz *et al.*, 1980). When pokeweed mitogen (PWM) induced Ig synthesis is used to assay for Con A-induced suppression, control as well as Con A-induced cells may exert inhibitory activity (Schwartz *et al.*, 1977; Haynes & Fauci, 1978; Lipsky *et al.*, 1978). This "spontaneous" suppressor activity of control cells is thought to reflect the expansion of a pre-existent inhibitory cell sub-population *in vitro* (Lipsky *et al.*, 1978).

In HD there is an increase in suppressor activity for proliferation induced by mitogens (Goodwin *et al.*, 1977; Twomey *et al.*, 1980; Schulof *et al.*, 1981) or cell bound alloantigens (Twomey *et al.*, 1975; Hillinger & Hertzig, 1978; Engleman *et al.*, 1979) which may contribute to the defect in cell-mediated immunity present in these patients (Twomey *et al.*, 1975; Schulof *et al.*, 1981). All these studies, however, have employed peripheral blood mononuclear cells (PB-MNC) and little information is available on the regulatory activity of tissue lymphocytes in this disease. It is clear that immunological changes occur in the spleen of HD patients prior to involvement with tumour (Payne *et al.*, 1976). In particular, manifestations of B-cell hyperactivity in the spleen (Longmire *et al.*, 1973; Payne *et al.*, 1976; Jones *et al.*, 1978) and bone marrow (Longmire *et al.*, 1974; Kass & Votaw, 1975) together with increased levels of polyclonal Ig in the serum of these patients (Landaas *et al.*, 1979) imply a defect of B-cell regulation in HD.

In this study the Con A-induced suppressor activity of splenic lymphocytes and PB-MNC from HD patients and control subjects for PWM-induced Ig synthesis was determined in an attempt to further investigate B-cell regulation in HD.

### Materials and methods

### Subjects

Fresh spleen tissue was obtained at staging laparotomy from 14 patients with HD, of which 3 showed histological evidence of disease involvement. In this group, 3 patients had lympho-

*Present address: Pediatric Hematology, Cornell University Medical Center, 1275 York Avenue, New York 10021, USA.
Correspondence: D.B. Jones
Received 12 September 1983; accepted 2 November 1983.

© The Macmillan Press Ltd., 1984

cyte predominant disease, 10 showed the nodular sclerosing pattern of HD and one patient had the mixed cellularity form. Clinically, stage Ia predominated in the uninvolved group. Patients with histological evidence of involvement in the spleen were staged as IIa or IIb.

Eight non-lymphomatous spleens showing normal histology were also obtained from patients incidental to abdominal surgery or from road accident cases, Heparinized venous peripheral blood was obtained from 5 of the HD spleen donors at the time of staging laparotomy. Normal heparinized venous peripheral blood was obtained from healthy laboratory personnel.

*Isolation of mononuclear cells*

Peripheral blood mononuclear cells (PB-MNC) were isolated by Ficoll/Triosil gradient sedimentation (Payne *et al.,* 1976). All tissue culture reagents were obtained from Gibco, Europe Ltd; (Paisley, Scotland). Cells were washed three times in calcium and magnesium free Hank's Balanced Salt Solution (HBSS-CMF) and resuspended in RPMI-1640 supplemented with penicillin (100 unit $ml^{-1}$), streptomycin (100 mg $ml^{-1}$), glutamine (20 mm) and 10% inactivated foetal bovine serum (FBS).

PB-MNC preparations usually contained 0–4% red blood cells, 0–2% granulocytes and 5–35% monocytes. Viability of the final mononuclear suspension trypan blue exclusion was >99%.

Fresh spleen tissue was minced in HBSS-CMF and filtered through a wire sieve. Splenic mononuclear cells were then isolated from this suspension as described for peripheral blood. Spleen mononuclear cell suspensions contained $89 \pm 3\%$ lymphocytes, $6 \pm 4\%$ non-specific esterase positive macrophages and $5 \pm 4\%$ polymorphonuclear leucocytes.

*Preparation of T-cell enriched and T-cell depleted MNC*

Splenic or peripheral blood MNC at $2 \times 10^6$ cells $ml^{-1}$ in HEPES buffered RPMI-1640 (HRPMI) were mixed with equal volumes of a 1% suspension of neuraminidase treated sheep red blood cells (SRBC) in HRPMI with 2% FBS. This suspension was incubated at 37°C for 10 min, centrifuged and re-incubated on ice for at least 2 h. T-enriched cells were prepared by further sedimenting the rosetting cells through a Ficoll/Triosil gradient, lysing the SRBC with TRIS buffered 0.83% ammonium chloride and washing with HBSS-CMF. T-depleted cells were recovered from the interface and washed in HBSS-CMF.

*Proliferative responses to mitogens*

Tissue or peripheral blood mononuclear cells ($2 \times 10^5$) in 0.2 ml of RPMI 1640 supplemented with 10% FBS were cultured in round-bottomed microtitre plates (Sterilin Ltd., Teddington, UK) at 37°C in an atmosphere of 5% $CO_2$ in air. PWM or Con A (10 $\mu$g) at a range of concentrations were added to triplicate cultures to obtain dose-response curves to these mitogens. After 3 days in culture, 0.2 $\mu$Ci of thymidine in 10 $\mu$l of medium (25 Ci $mmol^{-1}$, Radiochemical Centre, Amersham, UK) was added to each well. Between 18 and 24 h later the cells were harvested onto glass fibre filter paper (Whatman, Maidenhead, UK) using an automated harvester (Minivent, London, UK). Discs of air dried filter carrying deposited cells were placed in plastic scintillation insert vials (Sterilin, Teddington, UK) to which 0.5 ml of scintillation fluid was added. The radioactivity of the samples was measured in a Packard Tricarb liquid scintillation counter (model 544). Results were expressed as the mean counts per minute (cpm) of triplicate cultures.

Peripheral blood, normal spleen MNC showed peak responses to Con A when the final concentration of this mitogen in the culture was in the range of 5–20 $\mu$g $ml^{-1}$ and peak responses to PWM when $\sim$1/100 final dilution of this mitogen was used. In subsequent experiments, 10 $\mu$g $ml^{-1}$ and 20 $\mu$g of Con A was used to induce peripheral blood and splenic suppressor cells, while a 1/100 final dilution of PWM was used to stimulate Ig synthesis in the assay cultures. When T-enriched and T-depleted fractions of normal peripheral blood, normal spleen or HD spleen mononuclear cells were stimulated with 10 $\mu$g $ml^{-1}$ of Con A, suppressor activity for PWM-induced Ig synthesis was present in the T-enriched fractions with minimal suppression in the T-depleted cells. T-enriched fractions were therefore used to determine the Con A-induced suppressor activity of normal and HD spleens in order to standardize the populations under study.

*Induction of suppressor cells by Con A*

Cells ($5 \times 10^6$) from the prepared cell populations in 5 ml of RPMI 1640 supplemented with 10% FBS were incubated with 10 $\mu$g $ml^{-1}$ of Con A (Miles Laboratories Ltd., Slough, UK – batch number 180, cat. 79-003) for 18–24 h in an atmosphere of 5% $CO_2$ in air. Control cells were incubated without Con A. At the end of the culture period, control and Con A activated cells were washed twice in 3M-methyl-D-mannopyranoside (Sigma Chemical Company, Poole, Dorset, UK) and once in complete medium to remove cell-bound Con A then adjusted to $10^6$ cells $ml^{-1}$ in RPMI 1640 with

10% FBS. The viability of control and Con A activated cells was always >90% after washing.

*Responder cells*

PB-MNC from normal individuals were prepared in parallel with the washed cells from the primary culture. One or more of 6 normal volunteers were used as responders for the determination of suppressor activity in both control and HD patients, chosen as they showed high Ig production in response to PWM. The responder cells were adjusted to a final concentration of $10^6$ cells $ml^{-1}$ in complete RPMI 1640 with 10% FBS.

*Assay for suppressor cell activity*

Aliquots (0.15 ml) of the responder cell suspension ($10^6$ cells $ml^{-1}$) were added in triplicate to the wells of round bottomed microtitre plates (Sterilin Ltd., Teddington, UK). To these cells were added 0.05 ml of RPMI alone, control cells ($10^6$ cells $ml^{-1}$) or Con A-induced cells ($10^6$ cells $ml^{-1}$) giving a final volume of 0.2 ml in each well and a 3:1 responder to control or suppressor cell ratio in the co-cultures. Aliquots (0.2 ml; $10^6$ $ml^{-1}$) of control or Con A activated cells were also cultured alone to assess their contribution to the final Ig synthetic response. Each well received 11 $\mu$l of PWM (Gibco Biocult UK Ltd., batch A405109, cat. 670-5360) resulting in a 1/100 final dilution of the stock solution of this mitogen. The culture plates were incubated at 37°C in an atmosphere of 5% $CO_2$ in air for 7 days. On completion of this culture period, viable cell yields were measured and cytospin preparations made from triplicate wells. Good agreement was found when two different responders were used in the suppressor assay. The mean suppression was, therefore, determined when more than one responder was used in a single experiment.

*Immunofluorescent staining for intracellular Ig*

Cytospin preparations were fixed by immersion, in dry acetone at −20°C, stained with a polyvalent FITC-conjugated sheep anti-human Fab antibody (Tenovus Laboratory, Southampton, UK) and examined under UV illumination on a Leitz Orthoplan microscope. The percentage of cytoplasmic Ig positive cells ($CIg^+$) was established by counting at least 300 cells/slide. The number of cytoplasmic Ig positive cells per well ($CIg^+$/well) is calculated as follows:

$$CIg^+/\text{well} = \%CIg^+ \times \frac{\text{number of viable cells/well}}{100}$$

*Calculation of suppression*

Percentage suppression induced by Con A is calculated as follows:

$$1 - \left\{\frac{\text{mean } CIg^+/\text{well in Con A co-cultures}}{\text{mean } CIg^+/\text{well in control cultures}}\right\} \times 100$$

Percentage spontaneous suppression is calculated as follows:

$$1 - \left\{\frac{\text{mean } CIg^+/\text{well with responder cells alone}}{\text{mean } CIg^+/\text{well in control co-cultures}}\right\} \times 100$$

Statistical analyses were performed using the Mann-Whitney U test.

### Results

*Patterns of spontaneous and Con A-induced suppression of peripheral blood and spleen*

The addition of Con A activated T-enriched or unfractionated normal peripheral blood MNC to PWM stimulated responder cells suppressed Ig synthesis in comparison with control cells. Furthermore, the co-culture of control T-enriched unfractionated peripheral blood MNC with PWM stimulated responder cells also spontaneously suppressed Ig production in most cases as compared with the same number of responder cells cultured alone with PWM. The Con A induced suppressor activity is, therefore, measurable above the spontaneous suppressor activity.

When the spontaneous and Con A-induced suppression by T-enriched splenic MNC was studied (Tables I and II), it was found that control and Hodgkin's lymphoma patients showed two patterns of inhibition, indicated by Group 1 and Group 2 in the tables. Into Group 1 we have placed patients with higher apparent Con A-induced than spontaneous suppressor activity. Group 2 showed markedly higher spontaneous suppression in comparison with Con A-induced activity. There was no correlation between either Group 1 or Group 2 suppression and the age or sex of the patients. Furthermore, in the lymphoma patients, there was no relationship between histological type of disease, involvement of spleen with tumour, clinical stages of disease, presence of constitutional symptoms and either pattern of suppression.

**Table I** Group 1 spleens. Spleens showing lower spontaneous than Con A-induced suppression. HD spleen showing significantly higher spontaneous suppressor activity than normal ($P = 0.018$) as assessed by the Mann-Whitney U test.

| | | % Suppression | |
|---|---|---|---|
| *Name* | *Diagnosis* | *Spontaneous* | *Con A-induced* |
| DJ | Normal | 6.9 | 43.2 |
| LF | Normal | −47.9 | 59.0 |
| JB | Normal | −15.1 | 45.2 |
| MJ | Normal | 1.9 | 39.0 |
| MS | Normal | 60.7 | 74.1 |
| Mean | | 1.3 | 52.1 |
| RS | HD | 1.4 | 50.6 |
| CC | HD | −8.2 | 28.9 |
| UC | HD | −23.5 | 37.0 |
| GG | HD | 42.1 | 61.2 |
| MW | HD | 9.4 | 22.6 |
| EM | HD | 26.2 | 72.2 |
| DB | HD | 25.4 | 30 |
| Mean | | 10.4 | 43.2 |

**Table II** Group 2 spleens. Spleens showing higher spontaneous than Con A-induced suppression. HD spleen showing significantly lower spontaneous suppressor activity than normal ($P = 0.018$) as assessed by the Mann-Whitney U test.

| | | % Suppression | |
|---|---|---|---|
| *Name* | *Diagnosis* | *Spontaneous* | *Con A-induced* |
| RF | Normal | 75.4 | 21.7 |
| AW | Normal | 70.8 | 5.2 |
| PC | Normal | 49.4 | 7.2 |
| Mean | | 65.2 | 11.4 |
| DT | HD | 14.9 | −6.25 |
| KG | HD | 44.4 | −7.7 |
| DM | HD | 24.3 | 20.4 |
| DA | HD | 37.1 | −21.9 |
| DW | HD | 47.7 | 13.2 |
| Mean | | 33.7 | −2.3 |

### *Comparison of suppression in HD and control spleens*

In total the spontaneous and Con A-induced suppression mediated by the T-enriched cells of 8 normal spleens and a maximum of 14 HD spleens were studied (Figure 1). The mean spontaneous suppression for normal and HD spleen was 29.8%,

**Figure 1** Inhibition of the PWM induction of Ig containing cells by peripheral blood or splenic lymphocytes cultured alone (spontaneous) or with 10 or 20 μg Con A. Each point represents the mean of 3 determinations, negative values represent stimulation. (●) normal spleen; (▲) Hodgkin's disease spleen.

and 20.1% respectively. When splenic T-enriched cells were incubated with 10 $\mu g\,ml^{-1}$ Con A before co-culture with PWM stimulated responder cells a mean of 28.9% and 31.4% suppression of Ig synthesis resulted for normal and HD spleens respectively. There was no significant difference in spontaneous or Con A-induced suppression between the 2 spleen populations as assessed by the Mann-Whitney U test. Within the HD group, no correlation was observed between clinical stage of disease, A or B symptoms, histological type of disease or involvement of spleen with tumour and spontaneous or Con A-induced suppression.

### *Suppressor activity simultaneously measured in the peripheral blood and spleen of lymphoma patients*

Venous peripheral blood was obtained for 6 HD patients who were undergoing staging laparotomy. Simultaneous suppressor experiments were undertaken with cells from both blood and spleen. Insufficient volumes of HD peripheral blood were available to allow the T-enrichment of this population. The spleen T-enriched fractions contained a mean of 83.5% (range 78%–93.3%) while the unfractionated PB-MNC fractions contained 60.3% (range 48.6%–69.5%) of cells that rosetted with SRBC. Less than 1% of splenic T-enriched cells stained with non-specific esterase while a mean of 14.1% (range 7.5%–23.4%) of the unfractionated HD PB-MNC contained this enzymic activity. There were no significant differences in suppression between lymphoma peripheral blood and spleen (Figure 2). Individual patients, however, showed a large discrepancy in suppression between the two lymphoid compartments. The suppressor activity induced by 10 and 20 μg of Con A in the unfractionated PB-MNC of 5HD patients was significantly lower than that induced in identically prepared PB-MNC of the whole control group consisting of blood from 12 normal individuals ($P=0.05$). Normal and HD patients PB-MNC were regularly assayed in parallel on responders selected from the same donor panel. These data are presented in Figure 3. There was no significant difference in spontaneous suppressor activity measured in the blood, between control and HD patients. A mean of 61.5% (range 45.0%–72.5%) of the cells in the control group formed rosettes with sheep red blood cells before activation by Con A while the figures for HD patients was 60.3% (range 48.6%–69.5%). The mean percentage of cells that stained positively for non-specific esterase was 17.6% (range 10.1%–34.0%) in the control group and 14.1% (range 7.5%–23.4%) in the HD patients. The differences in suppression between controls and HD patients is, therefore, unlikely to be due to differences in the composition of the cell population under study.

It was not possible to obtain sufficient HD patient blood samples to enable correlation between suppression and clinical stage or histological type of disease.

**Figure 2** Spontaneous and Con A induced suppression of the PWM induction of Ig containing cells by lymphocytes from the blood and spleen of individual patients. Blood and spleen preparations from individual patients have the same symbol. Mean of 3 determinations, negative values represent stimulation.

**Figure 3** Spontaneous and Con A induced suppression of the PWM induction of Ig containing cells by lymphocytes from the blood of HD patients (▼) and normal controls (●). Mean of 3 determinations, negative values represent stimulation.

*Viable cell recovery after culture with PWM for 7 days*

Viable cell recoveries were measured after 7 days in the PWM assay. Table III shows the mean viable cell recoveries of the experimental groups studied. The number of cells present at the initiation of culture was $1.5 \times 10^5$ for the responder alone and $2.0 \times 10^5$ in co-cultures of responder and control or Con A-activated T-enriched cells. After 7 days, co-cultures containing Con A-activated cells consistently showed greater viable cell yields than co-cultures containing responder and control cells or cultures containing responder cells alone. Both control and Con A-activated T-enriched cells contained >1% of $GIg^+$ cells after culture alone with PWM for 7 days.

### Discussion

The majority of studies concerning Con A-induced suppressor activity in man have investigated peripheral blood mononuclear cells (Dwyer & Johnson, 1981). A few authors have described the ability of Con A activated splenic lymphocytes to suppress both Ig synthesis (Kraukauer *et al.*, 1980; Ilfeld & Kraukauer, 1982) and alloantigen induced proliferative responses (Sampson *et al.*, 1975). These studies did not compare blood and spleen. In this study splenic and peripheral blood MNC showed similar proliferative dose response curves to both PWM and Con A. Further, Con A induced suppressor activity was restricted to the T-enriched fractions of spleen cells, implying a common identity for the mitogen activated suppressor cell in both compartments. The levels of inhibition of Ig synthesis observed with both peripheral blood and splenic suppressor cells were similar.

Several authors have described a range of effects on PWM activation mediated by peripheral blood lymphocytes in culture, ranging from stimulation to marked suppression (Schwartz *et al.*, 1977; Haynes & Fauci, 1978; Lipsky *et al.*, 1978). Our results with T-enriched spleen cell fractions show that this is also the case in spleen. Spontaneous suppressor activity, attributed in the blood to the spontaneous activation of suppressor cells under culture conditions (Schwartz *et al.*, 1977) was apparent in mitogen-free cultures of cells from HD and control spleens. It is also clear from these results that there is a reciprocal relationship between spontaneous suppression and that induced by Con A, suggesting that in both cases the activated cell represents identical or overlapping populations.

Increased spontaneous suppression of proliferative responses has been found in HD (Twomey *et al.*, 1975; Goodwin *et al.*, 1977; Twomey *et al.*, 1980; Hillinger & Hertzig, 1978; Engleman, 1979) and may contribute to the defect in cell mediated immunity which is characteristic of patients with this disease. Con A-induced suppression of lymphocyte proliferation is either normal (Van Haelen & Fisher, 1981) or decreased (Schulof *et al.*, 1980). In this study we have determined the percentage of Ig synthesizing cells at the end of the culture period and the results may,

**Table III** Viable cell recovery

| Population under study | No. experiments | *Mean ± s.d. viable cell recovery* $\times 10^{-5}$ *Responder*[a] *alone* | *Responder*[a] *+ controls cells* | *Responder*[a] *+ Con A activated cells* |
|---|---|---|---|---|
| Unfractionated normal PB-MNC | 12 | 1.51±0.45 | 1.83±0.49 | 2.66±0.43 |
| T-enriched normal PB-MNC | 7 | 1.98±0.35 | 2.2 ±0.4 | 2.6 ±0.5 |
| T-enriched normal spleen MNC | 8 | 1.4 ±0.4 | 1.53±0.05 | 2.09±0.7 |
| T-enriched Non-HD lymphoma spleen MNC | 3 | 1.26 | 1.73 | 1.97 |
| T-enriched HD spleen MNC | 14 | 1.8 ±0.46 | 1.95±0.8 | 2.32±0.67 |

[a]Viable cell recovery after 7 days in culture with a 1/100 final dilution of PWM.

therefore, reflect the activity of a different population from those concerned with the inhibition of proliferation (Lobo & Spencer, 1979; Herscowitz *et al.*, 1980). It is clear from Tables I and II that in this system HD spleen preparations resemble the normal spleen pattern. When splenic suppressor populations were divided into those exhibiting low spontaneous suppression and those with natural activity (Groups 1 and 2 respectively), control and HD patients could be placed into both groups. Thus, although evidence of a defect in B-cell regulation exists in HD (Longmire *et al.*, 1973, 1974; Kass & Votaw, 1975; Payne *et al.*, 1976; Jones *et al.*, 1977) it was not possible to show a clear difference between control and HD patients using the Con A-induced system. In this respect it is interesting to note that Souhami *et al.* (1981), working with a specific antigen found evidence for a B-cell defect separate from the T-cell abnormalities present in this lymphoma. It is possible that the overall level of spontaneous suppression is lower in HD ($P=0.018$ in the Group 2) but the relatively small number of spleens in each group render this result difficult to substantiate.

In five cases it proved possible to determine the suppressor activity of both HD patients spleen and blood lymphocytes in parallel. As a group there was no difference between blood and spleen lymphocytes, though individual patients showed wide variation in the suppression observed in both compartments. Clearly, therefore, peripheral blood values for functional activity are not always representative of the tissue values in lymphoma. Further, whilst overall Con A suppression of PWM stimulated Ig synthesis was lower in HD blood than controls, as described by Schulof *et al.* (1980) for Con A suppression of proliferation, with the small sample available in this study we were unable to demonstrate a consistent sequestration of a particular subset of lymphocytes in the spleen in HD, (de Sousa *et al.*, 1977, 1978; Gupta & Tan, 1980).

The assay system employed the alteration of numbers of cytoplasmic Ig positive cells following PWM stimulation of PB MNC from a limited normal donor panel co-cultured with MNC from HD samples. Viable cell recovery (Table III) was never less in mitogen containing cultures than in controls. It is, therefore unlikely that apparent suppression was a result of cytotoxicity. Further, the comparison of figures for viable cell recovery for different control and experimental groups with those for the suppression obtained shows that dilution due to cell proliferating does not account for the observed reduction in Ig containing cells.

In conclusion, the data presented shows patterns of suppression of PWM-induced immunoglobulin synthesis in the spleen which resemble those described for peripheral blood. With the use of Con A to induce suppression and in contrast to other functions we have measured (Payne *et al.*, 1976; Al Sam *et al.*, 1982) we have not obtained convincing evidence of either the loss or sequestration of suppressor cells in HD spleens. It is also clear from the parallel studies undertaken that measurements of immune function in the blood in malignant lymphoma may be widely discordant from those obtained in relevant lymphoid organs, the data, therefore, indicates the importance of measuring more than one compartment in this type of study.

### References

AL SAM, S.Z., JONES, D.B., PAYNE, S.V. & WRIGHT, D.H. (1982). Natural killer (NK) activity in the spleen of patients with Hodgkin's disease and controls. *Br. J. Cancer*, **46**, 806.

DAMLE, N.K. & GUPTA, S. (1982). Heterogeneity in Concanavalin A-induced suppressor T-cells in man defined with monoclonal antibodies. *Clin. Exp. Immunol.*, **48**, 581.

DE SOUSA, M., YANG, M., LOPES-CORRALES, E. & 4 others. (1977). Ecotaxis the principle and its application to the study of Hodgkin's disease. *Clin. Exp. Immunol.*, **27**, 143.

DE SOUSA, M., TAN, C.T.C., SIEGAL, F.P., FILIPPA, D.A., TAN, R. & GOOD, R.A. (1978). Immunologic parameters in childhood Hodgkin's disease. II. T & B lymphocytes in the peripheral blood of normal children and in the spleen and peripheral blood of children with Hodgkin's disease. *Pediatr. Res.*, **12**, 143.

DWYER, J.M. & JOHNSON, C. (1981). The use of Concanavalin-A to study immunoregulation of human T-cells. *Clin. Exp. Immunol.*, **46**, 237.

ENGLEMAN, E.G., BENIKE, C., HOPPE, R.J. & KAPLAN, H.S. (1979). Suppressor cells of the mixed lymphocyte reaction in patients with Hodgkin's disease. *Transplant. Proc.*, **11**, 1827.

FAUCI, A.S., STEINBERG, A.D., HAYNES, B.F. & WHALEN, G. (1978). Immunoregulatory aberration in systemic lupus erythematosus. *J. Immunol.*, **121**, 1473.

GOODWIN, J.S., MESSNER, R.P., BANKHURST, A.D., PEAKE, G.T., SAIKI, J.H. & WILLIAMS, R.C. Jnr. (1977). Prostaglandin producing suppressor cells in Hodgkin's disease. *N. Engl. J. Med.*, **297**, 963.

GUPTA, S. & TAN, C. (1980). Subpopulations of human lymphocytes XIC. Abnormalities of T-cell locomotion and distribution of subpopulation of T and B lymphocytes in peripheral blood and spleen from children with untreated Hodgkin's disease. *Clin. Immunol. Immunopathol.*, **16**, 173.

HAYNES, B.F. & FAUCI, A.S. (1978). Activation of human B lymphocytes. VI. Immunoregulation of antibody production by mitogen-induced and naturally occurring suppressor cells in normal individuals. *Cell. Immunol.*, **36**, 294.

HERSCOWITZ, H.B., SAKANE, T., STEINBERG, A.D. & GREEN, I. (1980). Heterogeneity of human suppressor cells induced by Concanavalin A as determined in simultaneous assays of immune function. *J. Immunol.*, **124**, 1403.

HILLINGER, S.M. & HERZIG, G.P. (1978). Impaired cell mediated immunity in Hodgkin's disease mediated by suppressor lymphocytes and monocytes. *J. Clin. Invest.*, **61**, 1620.

ILFELD, D. & KRAUKAUER, R.S. (1982). Hydrocortisone reverses the suppression of immunoglobulin synthesis by Concanavalin A-activated spleen cell supernatants. *Clin. Exp. Immunol.*, **48**, 244.

JONES, D.B., PAYNE, S.V. & WRIGHT, D.H. (1977). Anti-lymphocytic globulin in Hodgkin's disease. *Biomedicine*, **27**, 177.

JONES, D.B., PAYNE, S.V. & WRIGHT, D.H. (1978). Absence of IgG lymphocytotoxins in untreated Hodgkin's disease patients. *Clin. Exp. Immunol.*, **34**, 100.

KASS, L. & VOTAW, M.L. (1975). Eosinophilia and plasmacytosis of the bone marrow in Hodgkin's disease. *Am. J. Clin. Pathol.*, **64**, 248.

KRAUKAUER, R.S., WALDMANN, T.A. & STROBER, W. (1980). Loss of suppressor T-cells in adult NZB/NZW mice. *J. Exp. Med.*, **144**, 662.

LANDAAS, T.O., GRIMMER, D., HEIER, H.E. & GODAL, T. (1979). Increased serum IgE in Hodgkin's disease is of polyclonal origin. *Acta Pathol. Microbiol. Scand.*, Section C, **87**, 377.

LIPSKY, P.E., GINSBERG, W.W., FINKELMAN, F.D. & ZIFF, M. (1978). Control of human B-lymphocyte responsiveness; enhanced suppressor T-cell activity after *in vitro* incubation. *J. Immunol.*, **120**, 902.

LOBO, P.I. & SPENCER, C.E. (1979). Inhibition of humoral and cell mediated immune responses in man by distinct suppressor cell systems. *J. Clin. Invest.*, **63**, 1157.

LONGMIRE, R.I., MCMILLAN, R., YELENOSKY, R., ARMSTRONG, S., LANG, J.E. & CRADDOCK, C.G. (1973). *In vitro* splenic IgG synthesis in Hodgkin's disease. *N. Engl. J. Med.*, **289**, 763.

LONGMIRE, R.L., MCMILLAN, R. & ARMSTRONG, S. (1974). Antibody-dependent lymphocytotoxicity induced by immunoglobulin-G from Hodgkin's disease splenic lymphocytes. *Proc. Am. Soc. Haematol.*, **17**, 163.

PAYNE, S.V., JONES, D.B., HAEGERT, D.G., SMITH, J.L. & WRIGHT, D.H. (1976). T and B lymphocytes and Reed-Sternberg cells in Hodgkin's disease lymph node and spleen. *Clin. Exp. Immunol.*, **24**, 280.

SAKANE, T., STEINBERG, A.D. & GREEN, I. (1978). Studies of immune functions of patients with systemic lupus erythematosus. *Arthrit. Rheum.*, **21**, 657.

SAMPSON, D., GROTELUESCHEN, C. & KAUFFMAN, H.M. (1975). The human splenic suppressor cell. *Transplantation*, **20**, 362.

SCHULOF, R.S., BRITON, J.L., MORTIMER, J.L. & 4 others. (1980). Concanavalin A-induced suppressor activity in Hodgkin's disease. *Clin. Immunol. Immunopathol.*, **16**, 454.

SCHULOF, R.S., BOCKMAN, R.S., GAROFALO, J.A. & 9 others. (1981). Multivariate analysis of T-cell functional defects and circulating serum factors in Hodgkin's disease. *Cancer*, **48**, 964.

SCHWARTZ, S.A., SHOU, L., GOOD, R.A. & CHOI, Y.S. (1977). Suppression of immunoglobulin synthesis and secretion by peripheral blood lymphocytes from normal donors. *Proc. Natl Acad. Sci.*, **74**, 2099.

SHOU, L., SCHWARTZ, S.A. & GOOD, R.A. (1976). Suppressor cell activity after Concanavalin A treatment of lymphocytes from normal donors. *J. Exp. Med.*, **143**, 1100.

SMITH, C.I. & SVEJGAARD, A. (1981). Concanavalin A-induced suppressor cells in normal individuals. *Scan. J. Immunol.*, **13**, 483.

SOUHAMI, R.L., BABBAGE, J. & CALLARD, R.E. (1981). Specific *in vitro* antibody response to Varicella Zoster. *Clin. Exp. Immunol.*, **46**, 98.

THORSBY. E. & BRATILIE, A. (1970). *A Rapid Method for Preparation of Pure Lymphocyte Suspensions in Histocompatibility Testing*. (Ed. Teraski), Copenhagen: Munksgaard, p. 655.

TWOMEY, J.J., LAUGHTER, A.H., FARROW, S. & DOUGLAS, C.C. (1975). Hodgkin's disease: an immunodepleting and immunosuppressive disorder. *J. Clin. Invest.*, **56**, 467.

TWOMEY, J.J., LAUGHTER, A.H., RICE, L. & FORD, R. (1980). Spectrum of immunodeficiencies with Hodgkin's disease. *J. Clin. Invest.*, **66**, 629.

VAN HAELEN, C.P.J. & FISHER, R.I. (1981). Increased sensitivity of lymphocytes from patients with Hodgkin's disease to Concanavalin A-induced suppressor cells. *J. Immunol.*, **127**, 1216.