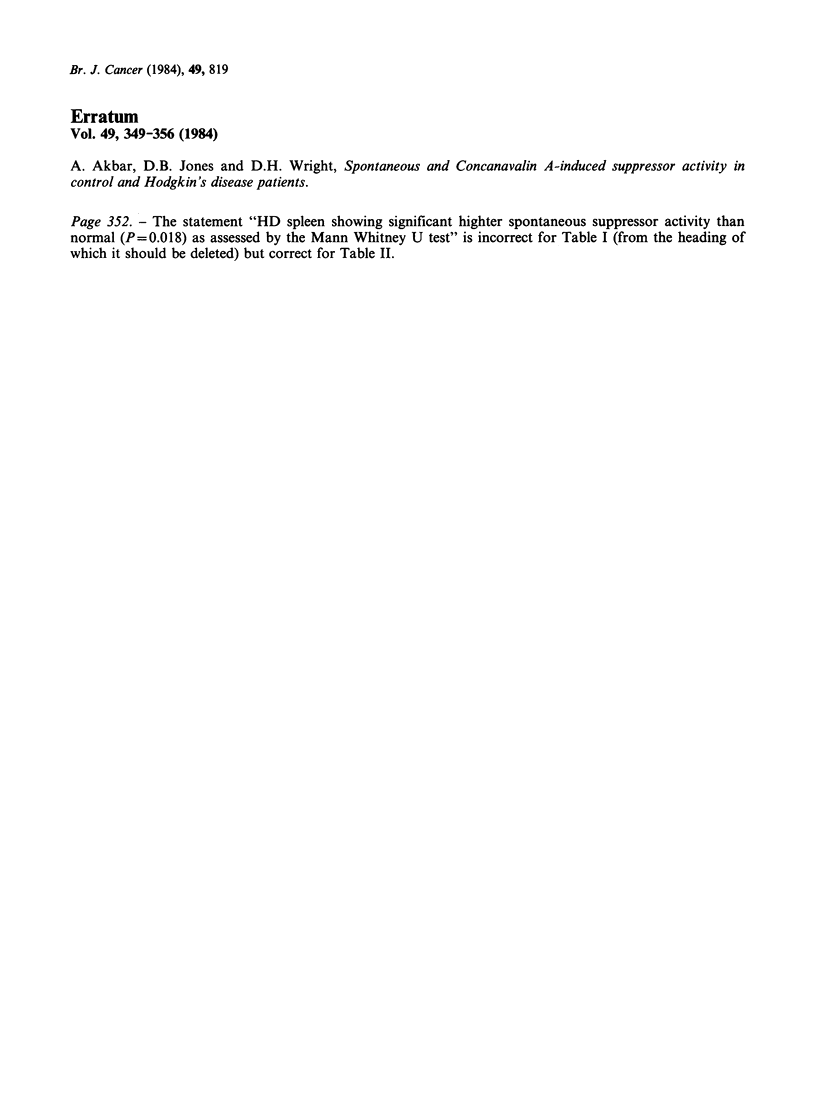# Erratum

**Published:** 1984-06

**Authors:** 


*Br. J. Cancer* (1984), **49**, 819

### Erratum
**Vol. 49, 349–356 (1984)**

A. Akbar, D.B. Jones and D.H. Wright, *Spontaneous and Concanavalin A-induced suppressor activity in control and Hodgkin's disease patients.*

*Page 352.* – The statement "HD spleen showing significant highter spontaneous suppressor activity than normal ($P = 0.018$) as assessed by the Mann Whitney U test" is incorrect for Table I (from the heading of which it should be deleted) but correct for Table II.